# Acquired Long QT Syndrome: Ventricular Fibrillation in an Otherwise Healthy Young Female

**DOI:** 10.7759/cureus.37263

**Published:** 2023-04-07

**Authors:** William P Ott, Shannay E Bellamy, Muzzamil Khan, Ahmad Shahid, Mohammad T Javed

**Affiliations:** 1 Internal Medicine, Jersey City Medical Center, Jersey City, USA; 2 Cardiology, Jersey City Medical Center, Jersey City, USA

**Keywords:** hypocalcemia complications, ssri side effects, cardiac arrythmia, ekg abnormalities, prolonged qtc

## Abstract

Long QT syndrome (LQTS) occurs when there is an abnormality of myocardial repolarization characterized specifically by a prolonged QT interval on an electrocardiogram (ECG). This can be particularly dangerous as it is associated with an increased risk of polymorphic ventricular tachycardia and a life-threatening arrhythmia otherwise known as torsades de pointes (TdP). We present a case of a 40-year-old Indian female whose medical history was significant only for anemia and depression/anxiety that presented in a ventricular fibrillation cardiac arrest after becoming dyspneic and light-headed while dancing. Of relevance, she was taking sertraline 50mg once daily, a class of medications known to prolong the QT interval as well as having low serum calcium on presentation. Both her initial and subsequent electrocardiograms illuminated significantly prolonged QTc intervals. She subsequently sustained a ventricular tachycardia cardiac arrest, which degenerated into ventricular fibrillation in the cardiac intensive care unit two days later. Ultimately, the patient was pronounced brain-dead by the end of the week. We concluded this to be a case of LQTS predisposing to TdP, which then would degenerate into ventricular fibrillation. This case highlights multiple risk factors that are known to predispose to the aforementioned etiology. Further research is needed not only on common medications and their dose-dependent relationship on the QT interval across different ethnic groups but also on educating providers regarding multiple risk factors they may or may not have the power to control.

## Introduction

Long QT syndrome (LQTS) occurs when there is an abnormality in myocardial repolarization characterized specifically by a prolonged QT interval on an electrocardiogram (ECG); it is associated with an increased risk of life-threatening arrhythmias such as polymorphic ventricular tachycardia and torsades de pointes (TdP) [1]. QT prolongation and ventricular fibrillation were first reported by Selzer and Wray as a result of the medication quinidine in 1964 [1]. Equally as importantly, less than three years later, Dessertenne articulated a polymorphic ventricular tachycardia where QRS complexes twist around an isoelectric line similar to a sinusoidal pattern; thereby creating the name torsades de pointes [2]. The most common etiology driving this phenomenon, excluding congenital or genetic defects, is medication therapy; therefore, it becomes a critical issue for healthcare providers as well as a sizable public health dilemma in regard to the wide array of medications that could potentially produce this adverse effect with the possibility of fatality [3]. The masses of patients who are potentially exposed to these drug therapies, in addition to our inability to accurately predict risk, make this dilemma even more worrisome. 

We present a case of a 40-year-old Indian female who was taking sertraline 50mg once daily, a class of medications known to prolong the QT interval as well as being hypocalcemic on presentation. Both her initial and subsequent electrocardiograms demonstrated significantly prolonged QTc intervals. This case highlights how acquired LQTS can be a misfortunate cumulation of etiologies that come together and exert their effect simultaneously, with the possibility of fatally disrupting one’s cardiac electrochemical gradients subsequently producing an arrhythmia. Following a literature review, it was found that the incidence of acquired LQTS is largely uncertain as most cases are underrecognized and underreported. In addition, most studies of LQTS were done on selected cohorts rather than population-based studies [1].

## Case presentation

This is a case of a 40-year-old Indian female with a medical history of anemia secondary to menorrhagia, anxiety, and depression who presented to the emergency room (ER) following cardiac arrest. She was noted to be at a dance studio earlier when she complained of dyspnea and sat down to rest and a few moments later, she lost consciousness. Emergency medical services (EMS) was called and on arrival, the patient was noted to be in cardiac arrest, and cardiopulmonary resuscitation was initiated with the patient being subsequently intubated in the field. The first rhythm noted during the resuscitation was ventricular fibrillation for which she was defibrillated at 360J. Resuscitation efforts continued for approximately 20 minutes, during which epinephrine was administered five times along with 300mg of amiodarone, which produced spontaneous circulation and ultimately was commenced on an epinephrine infusion. 

**Figure 1:**
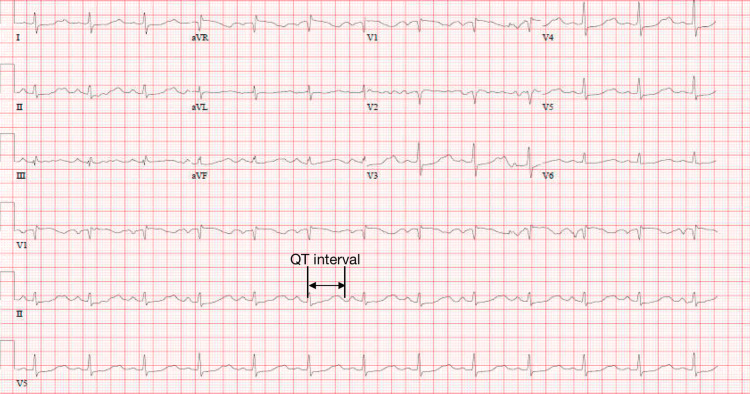
ECG on presentation illustrating sinus rhythm at 77 beats per minute with a prolonged QTc interval of 624ms (marked in black) with mild anterolateral depressions.

On arrival at the ER, she remained intubated and received ventilation via a bag-mask-valve device. She was then switched to mechanical ventilation while in the ER. She had a blood pressure of 81/33mmHg, a pulse rate of 77 beats per minute (Figure 1), and an oxygen saturation of 100% on mechanical ventilation with the fraction of inspired oxygen set to 100%. 

Physical examination was significant for an intubated female who was unarousable with bilateral dilated pupils which were slowly reactive to light. Corneal and gag reflexes were present, but she was breathing in synchrony with the ventilator on minimal sedation. Hematological investigations were significant for mild leukocytosis and microcytic anemia. Biochemistry was significant for hyperglycemia, with resultant hyponatremia, hypocalcemia, renal impairment, elevated liver transaminases, and a high-anion gap metabolic acidosis with significantly elevated lactic acid. Serum potassium and magnesium levels were within normal limits (Table 1).

**Table 1:** Laboratory investigations obtained on presentation Hb: hemoglobin; MCV: mean corpuscular volume; PT: prothrombin time; INR: international normalized ratio; PTT: partial thromboplastin time; Na: sodium, K: potassium, Cl: chlorine; CO2: carbon dioxide; Ca: calcium; BUN: blood urea nitrogen; AST: aspartate aminotransferase: ALT: alanine transaminase; ALP: alkaline phosphatase; TSH: thyroid-stimulating hormone

Laboratory test on admission	Result	Normal Range
WBC	13.6 K/UL	4.5-11.0K/UL
Hb	7.7 g/dl	14-18g/dL
MCV	78.3 fL	80.0-100.0 fL
Platelets	200 K/UL	130 – 400K/UL
PT	11.3 sec	12-15.1 sec
INR	0.97	0.85-1.14
PTT	61.7 sec	25.4-36.7 sec
Serum Glucose	605 mg/dL	74-106 mg/dL
Na	133 mmol/L	136-145 mmol/L
K	3.6 mmol/L	3.5-5.1 mmol/L
Cl	97 mmol/L	98-107 mmol/L
CO2	12 mmol/L	20-31 mmol/L
Ca	7.5 mg/dL	8.7-10.4 mg/dL
Ionized Calcium	3.6 mg/dL	4.8-5.6 mg/dL
Magnesium	2.26 mg/dL	1.60-2.60 mg/dL
Phosphorus	4.0 mg/dL	2.4-5.1 mg/dL
BUN	18 mg/dL	9-23 mg/dL
Creatinine	1.73 mg/dL	0.70-1.30 mg/dL
AST	606 Units/L	8-34 Units/L
ALT	521 Units/L	10-49 Units/L
ALP	104 Units/L	46-116 Units/L
Albumin	3.7 g/dL	3.2-4.8 g/dL
Lactic Acid	>12.20 mmol/L	0.50-1.99 mmol/L
TSH	3.14 mIU/L	0.40-4.50 mIU/L
High Sensitivity Troponin	66.2 ng/L	3.0-34.0 ng/L

**Figure 2:**
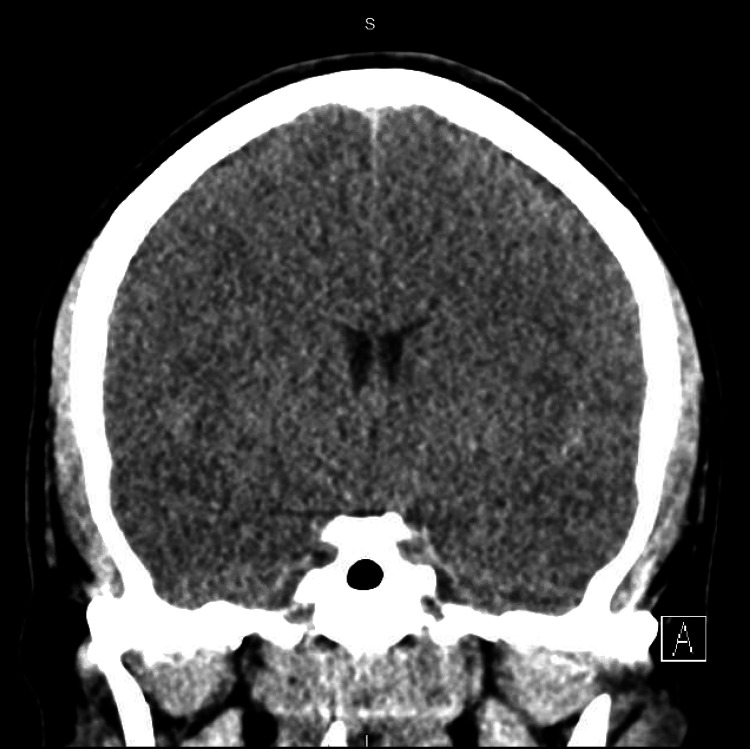
CT Head on presentation demonstrating loss of definition of the ventricular grey-white junction. The sulci are effaced as is the ventricular system. These findings are consistent with diffuse cerebral edema

Urinalysis showed moderate blood with red blood cells of 36 per high-powered field (hpf) and white blood cells of 43 per hpf without the presence of nitrites or leukocyte esterase. The urine drug screen was negative. Chest radiograph on admission confirmed endotracheal tube placement but did not show any infiltrates or pneumothorax. Computed tomography of the head showed diffuse cerebral edema thought to be sequelae of hypoxemia (Figure 2). 

An echocardiogram done on admission showed a moderately reduced left ventricular (LV) systolic function with an LV ejection fraction of 35%. Right ventricular systolic function was normal. Additional history from her husband at that time revealed no prior cardiac history. He reported that she had occasionally complained of chest pain and palpitations over the preceding year which she had attributed to her anxiety. She had been taking sertraline 50mg once daily without any noted adverse effects. She had no family history of cardiac disease or sudden cardiac death. Her husband reported that she had been following up with a primary care physician and her most recent visit was four months prior. Records obtained from that visit were significant only for microcytic anemia. There were no prior ECGs available. 

**Figure 3:**
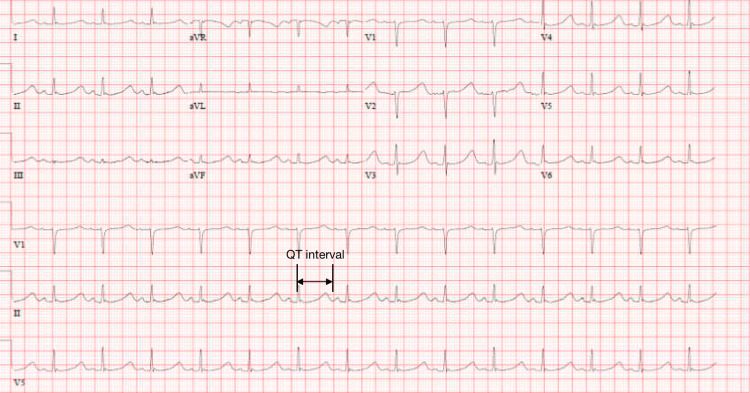
ECG on the morning following presentation in sinus rhythm at 86 beats per minute and a prolonged QTc interval (marked in black) of 615ms with resolution of the anterolateral depressions

The patient was admitted to the cardiac intensive care unit and underwent hypothermia protocol and remained intubated, although hemodynamically unstable, requiring vasopressor support. Subsequent ECGs continued to show sinus rhythm with prolonged QT (Figure 3).

**Figure 4:**
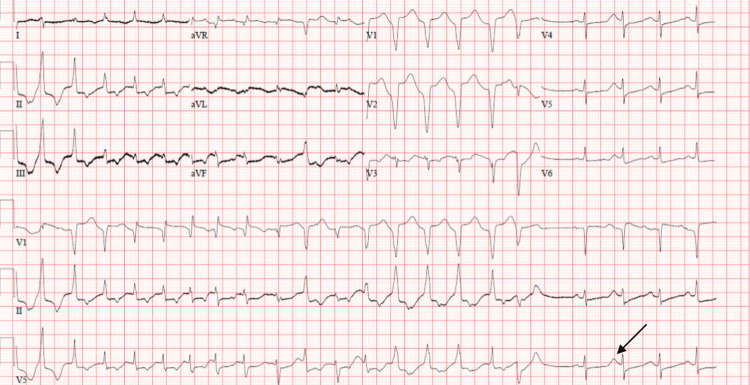
ECG illustrating atrial fibrillation with rapid ventricular response in addition to premature ventricular complexes before breaking into sinus rhythm with a prolonged QTc interval of 607ms as seen by the P wave buried within the T wave (marked with arrow) after the first beat in return to sinus

On the evening of her second day of admission, the patient had another cardiac arrest. She had begun the rewarming phase of the hypothermia protocol approximately nine hours prior. The patient had ventricular tachycardia observed on the telemetry monitor prior to going pulseless into cardiac arrest. During the cardiopulmonary resuscitation, she was defibrillated on two occasions for ventricular fibrillation before the return of spontaneous circulation. This time again, she had a total cardiac arrest time of approximately 20 minutes. A lidocaine drip was initiated. The ECG obtained following the cardiac arrest is shown in Figure 4.

Over the subsequent 48 hours, she showed no improvement in neurological function and was noted to have absent brainstem reflexes. She exhibited biochemistry and urine studies suggestive of hypernatremia as a result of central diabetes insipidus, which was treated and slowly corrected via desmopressin administration. Video electroencephalogram displayed no awake or sleep features, profound diffuse slowing, and attenuation of background without epileptic or periodic rhythmic discharges, consistent with profound encephalopathy and deemed likely to be hypoxic-ischemic in etiology. Nuclear medicine brain flow study on day five of admission would ultimately illustrate findings consistent with brain death. Additionally, bedside apneic and caloric oculovestibular reflex testing both indicated brain death. After several palliative discussions with the patient’s husband and extended family members, the decision was made to withdraw any further medical care. A cardiac catheterization, though, which was conducted as a requirement prior to organ donation would ultimately reveal unremarkable coronary arteries. 

## Discussion

A prolonged QT is defined by a QTc of more than 470msec in males and more than 480msec in females [4]. LQTS is particularly dangerous as it is associated with an increased risk of polymorphic ventricular tachycardia and a life-threatening arrhythmia otherwise known as TdP [4]. TdP typically has a rate of 160 to 250 beats per minute with a cyclic alteration of the QRS axis through 180 degrees every five to 20 beats [4]. It is typically short-lived and terminates spontaneously, but most patients will experience multiple episodes of this arrhythmia [4-5]. 

LQTS can be either congenital or acquired, but there is potential overlap between these two etiologies, as some with acquired LQTS can have underlying pathogenic variants which do not meet all the clinical criteria for congenital LQTS [4]. In these individuals, the alteration in repolarizing currents is insufficient to prolong the QT interval at rest. This may be due to redundancy in repolarizing currents (called repolarization reserve) [4]. Acquired LQTS typically results from drug therapy, although a number of patient and medication-related factors may enhance the risk of drug-induced LQTS such as electrolyte derangements, eating disorders, coronary artery disease, and bradyarrhythmias [4]. When medications are implicated in LQTS, they almost universally do so by blocking the "IKr" current, which is mediated by a potassium channel in myocardial cells. This potassium channel is encoded by the KCNH2 gene [6]. 

One of the most prevalent risk factors for drug-induced TdP is female gender [4]. Between 55% and 70% of people with drug-induced tdP are female, regardless of whether it is caused by a cardiac or noncardiac medication [4,7,8]. Compared with males, females naturally have a longer QTc, lower repolarization reserve, and a higher risk of TdP with drugs that even mildly block IKr [9]. Some of this may also be exacerbated by the fact that sex steroids may affect ion channel expression, leading to sex differences in the QT interval [10]. We hypothesize that an overlap of partially both congenital and acquired via medication-induced prolonged QT syndrome occurred in our case. Our patient may have had a silent genetic predisposition to being more sensitive to the potentially QT-prolonging effects of sertraline and her moderate hypocalcemia of 7.5mg/dL (corrected 7.7mg/dL) seen on presentation. Her reported history of short-lived anxiety attacks and ultimately her sudden dyspnea may have been symptomatic signs of her active incidents of ventricular arrhythmia. 

The incidence of acquired LQTS is uncertain largely due to most available studies relying on selected cohorts rather than population-based studies [4]. Medication-related acquired LQTS may underlie a sizable proportion of sudden cardiac death (SCD) in the United States. For example, in a study of 525 autopsy-confirmed, non-traumatic sudden deaths from San Francisco County, over half of the individuals had been taking at least one QT-prolonging medication. Additionally, one-third of people who had experienced a drug overdose as a cause of SCD were taking a QT-prolonging medication [11]. Our patient was taking a selective serotonin reuptake inhibitor (SSRI) daily, although sertraline compared to citalopram/escitalopram may be considered low risk for QT prolongation [12], even in tandem with her risk factors of being a female, hypocalcemic, and possibly even with genetic polymorphisms. We postulate each item played an important role in her multifactorial origin. 

## Conclusions

LQTS, whether congenital or acquired, can have devastating consequences on unsuspecting individuals. Both of these subtypes are understood to be likely underreported owing largely in part to being underrecognized. Difficulty arises, especially in regards to congenital variations of this syndrome, due to variable levels of penetrance exerted. Further research is needed not only on common medications and their dose dependent relationship on the QT interval across ethnic groups, but also on educating providers regarding multiple risk factors they may or may not have the power to control in their patients. This case highlights how acquired LQTS can be a misfortunate cumulation of etiologies that come together and exert their effect simultaneously, just enough at times, to fatally disrupt one’s cardiac electrochemical gradients and subsequent rhythm. 
